# Origin of a Second School

**Published:** 1835

**Authors:** 


### Origin of a second School.

The proceedings of which we have given a documentary narrative, were originated and prosecuted, by a Board of Trustees, most of whom had *not* been designated to the General Assembly, by the friends of College Reform; they were indeed, appointed on the last day of the late session, and taken in some degree hap-hazard. Thus they were not *the* Board of the reformers; but a compromise between the reformers and conservatives in the Legislature. Still, it is well known, that a decided majority of them were of *opinion*, that extensive changes in the Faculty of the College, were indispensable to its revival and future usefulness; and we do not entertain a doubt, that if the professors had resigned and taken their chance for re-election, a new and able faculty would have been made up. We have seen that this was not done, and that by the effort at reorganization, the College was unexpectedly brought into a condition, that seemed likely to reduce its next class still lower than the last, if not, indeed, for a year to close the doors. Thus as new institutions were starting up in different towns of the West, there was the utmost danger, that Ohio would lose the opportunity of perpetuating a good medical school, although she had been the second state, in the Valley of the Mississippi, to commence medical instruction; and had given more public money to the object, than all the Western states besides. Now there were citizens of Ohio, both in and out of the profession, who could not but feel great inquietude at this prospect; and they believed it a duty to prevent if possible, the loss to the state and city, of what had been expended by the former within the latter, on this important object. It was *this desire*, that gave birth to a new school. They in whom it existed, thought that if some other corporation, would take up the subject of reform, it might ultimately be accomplished, and a single good school secured to the state.

In this exigency, the Cincinnati College offered a resource, and it was forthwith drawn upon. A general meeting of the Board of Trustees, 20 in number, was called, and two thirds obeyed the summons. Of this Board, a fifth part reside in the country, and several are physicians. The meeting having been organized, Dr. Joshua Martin, of Xenia, offered the following preamble and resolutions:

"Whereas the recent attempt of the Medical profession and the General Assembly of Ohio to reorganize and improve the condition of the Medical College of Ohio, have, as we are informed, been unsuccessful, (the Board of Trustees of said College, having adjourned *sine die*, leaving two or three of its professorships vacant) and whereas there is the utmost danger, that Ohio will lose the advantages of a Medical institution, unless immediate measures be taken to organize a substitute for said College; therefore be it

*Resolved*, That this Board will proceed forthwith to establish a medical department of the Cincinnati College.

This preamble and resolution were on motion of Mr. Morris, referred as follows: "*Resolved*, That the preamble and resolution submitted by Dr. Martin, be referred to a committee of five members, with instructions to inquire into the expediency of the proposed measure and report to the Board the most suitable mode of carrying said resolution into effect."

Whereupon Dr. Joshua Martin, Ephraim Morgan, Albert Picket, Dr. William Mount, and William R. Morris were appointed that committee.

At the next meeting of the Board, the committee submitted the following report:

"The committee to whom was referred the consideration of the expediency of establishing a medical department in the Cincinnati College, and the best mode of carrying the same into effect, have had the subject under consideration, and beg leave to report to the Board—

That your committee are satisfied that from the peculiar situation in which the Medical College of Ohio is placed at this time, the interests of the state and especially of this community require, that this Board should immediately create a medical department, and appoint a medical faculty; and your committee, therefore, recommend that the following chairs be created to be filled by the persons whose names are respectively attached."

In making their appointments, the Board acted with fidelity to the principle on which they had started. Selecting Dr. Eberle, Dr. Cobb and Dr. Gross from the Medical College of Ohio, they united with them, in the completion of the Faculty, no gentleman who had not been strongly recommended to the Board of Trustees of that institution, by its own committee; and thus sought (to repeat the expression of an idea) to finish what the trustees of that institution had begun, but had been unable to accomplish, although an actual majority of the Board were in its favor.

Thus it was not the design of the Cincinnati College to set up a permanent rival, for their professors were at liberty to resign, and the whole of them, had they all accepted, might by a simple resolution of the trustees of the Medical College of Ohio, be transferred to that institution. At the same time, it was and is the determination of the trustees of the former, as we have intimated in the preceding article, to render their medical faculty permanent and respectable unless it should be invited into the other institution and wish to go. Their corporate powers are those of a university, as will appear from the following extract from their charter, granted in the same month and year, with that of the Medical College of Ohio:

"Sec. 6. *Be it further enacted*, That the Board of Trustees of the said College may grant and confer on any candidate, in such form as they may direct, all or any of the degrees that are usually conferred in any College or University within the United States."

They had in charge an ample and commodious edifice, in the centre of the city, which can be advantageously appropriated to the accommodation of pupils; and they saw and felt, that unless something should be immediately and effectually done, in the establishment of a medical school on sound principles and with the requisite number of professors, there would in all probability be no public medical instruction in Cincinnati next winter, and the pupils of Ohio, be under the necessity of visiting distant universities or of foregoing the advantages which they confer.

It may be proper here to remark, that the medical schools of the United States, generally, are departments of Colleges and Universities, designed for instruction in literature and science, at large, like the Cincinnati College; and that the Medical College of Ohio is perhaps the only separate and independent medical school in the United States.

Such were the patriotic motives which led them to engage in this enterprize, and will hold them to its unwavering prosecution, unless a better policy should arise in the Board of the Medical College, and those whom they have appointed, be provided for in that institution. To this transfer they have, in their individual and private character, constantly expressed a perfect willingness; for they have no personal interest in maintaining their new department; and are the friends and neighbors of the trustees of the Medical College of Ohio. Still having put their hand to the plough, they will not look back; but have evidently resolved, to persevere until the great object of establishing in Cincinnati, a Medical school, that shall be worthy of the state of Ohio, creditable to the city, and beneficial to the West, shall be fully accomplished.

---

